# Beat-by-Beat Estimation of Hemodynamic Parameters in Left Ventricle Based on Phonocardiogram and Photoplethysmography Signals Using a Deep Learning Model: Preliminary Study

**DOI:** 10.3390/bioengineering11080842

**Published:** 2024-08-19

**Authors:** Jiachen Mi, Tengfei Feng, Hongkai Wang, Zuowei Pei, Hong Tang

**Affiliations:** 1 School of Biomedical Engineering, Dalian University of Technology, Dalian 116024, China; jiachenmi@dlut.edu.cn (J.M.); tengfeifeng@dlut.edu.cn (T.F.); wang.hongkai@dlut.edu.cn (H.W.); 2 Liaoning Key Lab of Integrated Circuit and Biomedical Electronic System, Dalian University of Technology, Dalian 116024, China; 3 Dalian Key Laboratory of Digital Medicine for Critical Diseases, Dalian 116024, China; 4 Department of Cardiology, Central Hospital of Dalian University of Technology, No.826 Xinan Road, Dalian 116033, China; peizuowei@dlut.edu.cn

**Keywords:** hemodynamic parameters, phonocardiogram, photoplethysmography, deep learning model

## Abstract

Beat-by-beat monitoring of hemodynamic parameters in the left ventricle contributes to the early diagnosis and treatment of heart failure, valvular heart disease, and other cardiovascular diseases. Current accurate measurement methods for ventricular hemodynamic parameters are inconvenient for monitoring hemodynamic indexes in daily life. The objective of this study is to propose a method for estimating intraventricular hemodynamic parameters in a beat-to-beat style based on non-invasive PCG (phonocardiogram) and PPG (photoplethysmography) signals. Three beagle dogs were used as subjects. PCG, PPG, electrocardiogram (ECG), and invasive blood pressure signals in the left ventricle were synchronously collected while epinephrine medicine was injected into the veins to produce hemodynamic variations. Various doses of epinephrine were used to produce hemodynamic variations. A total of 40 records (over 12,000 cardiac cycles) were obtained. A deep neural network was built to simultaneously estimate four hemodynamic parameters of one cardiac cycle by inputting the PCGs and PPGs of the cardiac cycle. The outputs of the network were four hemodynamic parameters: left ventricular systolic blood pressure (SBP), left ventricular diastolic blood pressure (DBP), maximum rate of left ventricular pressure rise (MRR), and maximum rate of left ventricular pressure decline (MRD). The model built in this study consisted of a residual convolutional module and a bidirectional recurrent neural network module which learnt the local features and context relations, respectively. The training mode of the network followed a regression model, and the loss function was set as mean square error. When the network was trained and tested on one subject using a five-fold validation scheme, the performances were very good. The average correlation coefficients (CCs) between the estimated values and measured values were generally greater than 0.90 for SBP, DBP, MRR, and MRD. However, when the network was trained with one subject’s data and tested with another subject’s data, the performance degraded somewhat. The average CCs reduced from over 0.9 to 0.7 for SBP, DBP, and MRD; however, MRR had higher consistency, with the average CC reducing from over 0.9 to about 0.85 only. The generalizability across subjects could be improved if individual differences were considered. The performance indicates the possibility that hemodynamic parameters could be estimated by PCG and PPG signals collected on the body surface. With the rapid development of wearable devices, it has up-and-coming applications for self-monitoring in home healthcare environments.

## 1. Introduction

Monitoring left ventricular hemodynamic parameters has significant clinical value for cardiovascular diseases (CVDs), such as the monitoring of critically ill patients’ states, chronic heart failure observation, and myocardial ischemia diagnosis [1,2,3,4]. Previous studies have demonstrated that hemodynamic changes prior to the onset of symptoms and continuous hemodynamic monitoring can help to identify early clues of dysfunction [5,6]. Left ventricular blood pressure (LVBP), which is an important indicator for assessing cardiac function, contains many hemodynamic parameters [7]. Left ventricular systolic blood pressure (SBP) can be seen to rise in hypertensive patients and the condition of aortic valve stenosis [8,9]. Anomalies in left ventricular diastolic blood pressure (DBP) can be observed in patients with left heart failure, restrictive cardiomyopathy, and others [10]. The maximum rate of left ventricular pressure rise (MRR, LV + dP/dtmax) and maximum rate of left ventricular pressure decline (MRD, LV − dP/dtmax) are common, robust, and sensitive indexes of changes in ventricular contractility and diastolic capacity, respectively [7,11]. 

Existing non-invasive measurement methods are not sensitive enough to detect hemodynamics variation for early treatment [6]. For example, ultrasound, which is a useful tool for monitoring hemodynamic parameters in critically ill patients, can be used to reflect ±dP/dt only when mitral regurgitation exists and cannot be used to evaluate the isovolumic contraction state [12,13]. SBP, DBP, and other blood pressure (BP) parameters are always determined via cuff devices in clinical settings or intra-artery catheters on critically ill patients and need operation from specialist nurses [2,14]. This measurement provides a poor experience for long-term, continuous BP monitoring [6]. Other imaging modalities, such as MRI, echocardiography, and nuclide scanning, are expensive and hard to implement. Left ventricular hemodynamic parameters can be measured accurately via invasive catheters in hospital operating rooms, but they are high-cost and inconvenient [15]. Thus, portable and low-cost methods for non-invasive intraventricular hemodynamics monitoring need to be developed to give early warnings of cardiovascular diseases even through self-monitoring at home.

As useful diagnostic assistants for checking cardiac pathology, auscultations of phonocardiogram (PCG) signals and photoplethysmography (PPG) signals are easy to implement at a low cost. Many studies have indicated that there are inherent relations among PCG signals, PPG signals, and BP. Shah et al. [16] and Sakamoto et al. [17] revealed that there were linear relationships between the rising rate of left ventricular BP and the amplitude of the first heart sound in the 1960s. Refs. [14,18,19] extracted multi-domain features from PCG signals to estimate SBP and DBP, showing high correlation coefficients. Tang et al. [20] studied the relationship between SBP and different pulse arrival times (PATs). Refs. [2,21,22,23] predicted BP parameters using PPG signals, and most of the good performances from these parameters met the AAMI standard. Marzorati et al. [15] described an integrated chest-wearable apparatus for continuous blood pressure measurement by recording PPGs and PCGs, which showed a promising application of hemodynamic parameter monitoring algorithms based on PPGs and PCGs.

In recent years, the performance of both regression and classification tasks in different fields, such as speech recognition and machine translation, has been significantly improved with the rapid development of deep learning [24]. Additionally, there are more and more studies about biomedical signals focusing on deep neural networks [25]. Refs. [26,27,28] showed that a higher performance for arrhythmia classification could be achieved via different deep learning architectures than classification from experts. Heart sound classification for CVDs [29] and epileptic seizure detection using EEGs [30,31] based on deep learning models were also studied sufficiently. Refs. [2,32,33] described some applications of deep learning on different BP prediction assignments. Therefore, a deep learning model developed for hemodynamic parameter estimation is an encouraging attempt.

After having investigated the previous literature, some issues still need to be considered, though there has been excellent work in the previous studies completed. (1) To the best of our knowledge, few studies for estimating hemodynamic parameters used intraventricular BP values, which are more sensitive to CVDs. (2) Most studies focused on extracting multi-domain features and estimated only one hemodynamic parameter, which was not an end-to-end architecture and had a low ability for real-time monitoring. (3) The BP ranges used in previous studies were not large enough to learn about the relations between PCG, PPG, and hemodynamic parameters.

Considering the above aspects, an end-to-end deep learning model that uses raw data of PCG and PPG signals as inputs is built to estimate four intraventricular hemodynamic parameters in this study. The maximum rate of left ventricular pressure rises (MRR) and the maximum rate of left ventricular decline (MRD), which have seldom been estimated in previous studies as non-ventricular BP does not effectively capture rapid changes, are considered besides SBP and DBP. In this study, data were collected from three beagle dogs who were injected with different doses of epinephrine to generate a large range of BP. PCGs on the chest, blood pressure measured by an invasive catheter inserted into the left ventricle, PPGs over the femoral artery, and ECGs were simultaneously recorded. The number of cardiac cycles was over 12,000, which is sufficient for deep learning. As most studies were less convincing due to a random split scheme that ignores the similarity between nearby cardiac cycles or segments, we used a 3-1-1 training, validation, and testing scheme using different records for fivefold cross-validation. The performance indicated that left intraventricular hemodynamic parameters could be estimated with PCG and PPG signals, and there are promising applications for non-invasive intraventricular hemodynamic parameter monitoring. Estimation of hemodynamic parameters across subjects was also studied in this work, which showed that errors were mostly caused by individual differences, and the model could be calibrated to some extent by adding some individual information.

## 2. Materials and Methods

### 2.1. Data Acquisition and Preprocessing

**Figure 1:**
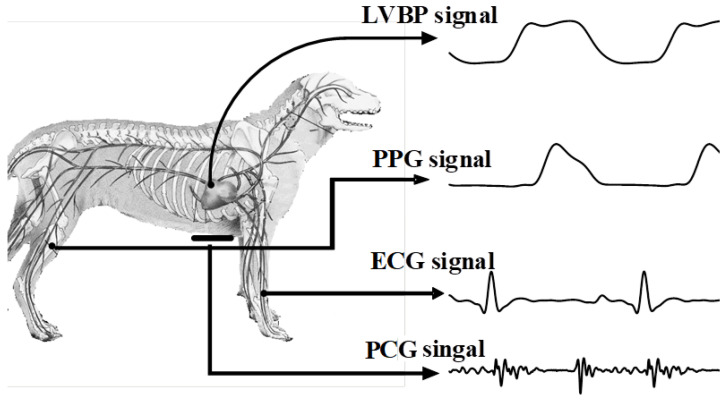
Data collection scheme of the experiment.

The experiment was approved by the Animal Care Committee of Chongqing Medical University and conducted on three healthy beagle dogs weighing 9–10 kg. This animal study was approved by the Ethical Committee of Dalian University of Technology under approval number DUT20141105_001. The procedure of the experiment was as follows: First, the dogs laid down calmly in the supine position and were anesthetized with xylazine (0.2 mL/kg) before recording data. A catheter filled with a heparinized solution (500 units/mL) was inserted into the left ventricle via the carotid artery. Then, different doses of epinephrine (0.5 g/kg, 1 g/kg, or 2 g/kg) were administered to the dogs via an intravenous infusion route with 0.9% saline to produce a large range of blood pressure levels. Data were collected from 10 s before injection of epinephrine until BP values returned to normal. The procedure was repeated 3–5 times. As Figure 1 shows, ECGs, PCGs, PPGs, and BP were recorded synchronously during the whole procedure. BP was recorded by a pressure transducer (MLT0699, ADInstruments, Dunedin, New Zealand) which was connected to the inserted catheter and calibrated at the standard atmospheric pressure. The ECG electrodes were placed on both of the dog’s forelimbs as ECG lead I (PL3508, PowerLab 8/35, ADInstruments, Bella Vista, Australia). A microphone transducer (MLT201, ADInstruments, Australia) was placed at the apex of the heart to record external PCGs. PPGs were recorded non-invasively by a photoplethysmogram sensor (MLT1020FC, ADInstruments, Australia) which was affixed to the femoral artery. The digital sampling frequency of all signals was 1 kHz (MP150, BIOPAC, Goleta, CA, USA). In the end, 41 records were obtained. The details of the records are shown in Table 1.

**Figure 2:**
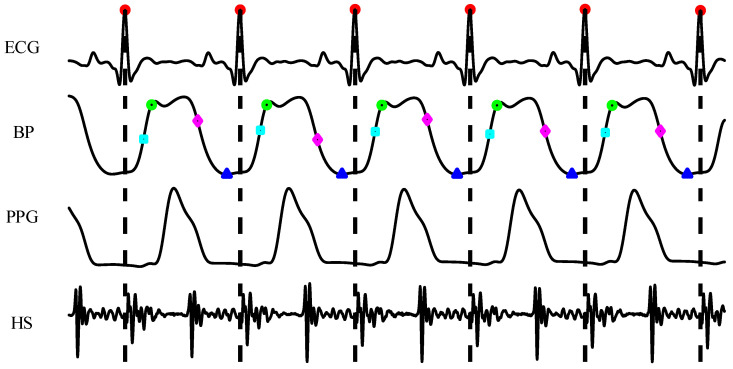
Scheme of splitting signals into cardiac cycles based on R-wave locations.

After collecting the experimental data, pre-filters were used to improve the signal quality. The passbands of the pre-filters were set as follows: [30 200] Hz for PCGs, [0.5 40] Hz for ECGs, and [0.5 20] Hz for PPGs and blood pressure. Zero-phase filtering technology was used for these filters to avoid time delay. The Pan–Tompkins (P&T) algorithm [34] was used to detect R-waves of the ECGs. Then, PCG, PPG, and BP signals were segmented into cardiac cycles according to the location of R-waves, as shown in Figure 2. Cycles with bad PCG signal quality or bad PPG quality were automatically removed by algorithms. SBP was acquired by detecting the BP value at the end of the systolic stroke. The minimum values of BP in the diastolic phase were detected as the DBP. The maximum and minimum values of the BP’s first-order derivative were extracted as the MRR and MRD, respectively. Abnormal hemodynamic parameters whose difference values exceeded three standard deviations from the mean were fixed using nearby values. Finally, 12,328 cardiac cycles were acquired after preprocessing. For subject 1, 7529 cardiac cycles were acquired with high-quality signals. From subject 2, 3935 cycles were extracted. Due to the low signal-to-noise ratio, only 864 cardiac cycles were extracted from subject 3. 

### 2.2. Hemodynamic Parameter Estimation Model

**Figure 3:**
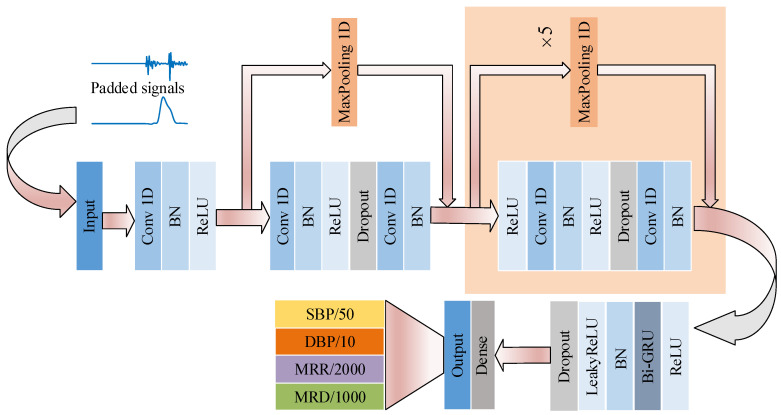
The structure of the end-to-end model for estimating hemodynamic parameters. The inputs are PCG and PPG signals, which are padded to 1s, and the outputs are hemodynamic parameters, which are scaled by different weights to have the same contribution to the loss function.

Hemodynamic parameter estimation can be seen as a time-series regression problem. Feature extraction is an important step in the regression task of hemodynamic parameter estimation. The performance of regression is influenced by the quantity of the extracted features. In recent years, without hand-crafted features, deep learning models with end-to-end architectures have shown excellent performances for both classification work and regression work in many fields [26,35]. In this study, a 64-layer end-to-end model for hemodynamic parameter estimation was built to learn features from PCG and PPG signals and estimate hemodynamic parameters. The details of the model are illustrated in Figure 3.

In the first part of the model, several residual convolutional modules with reference to the model used in [26] are used to learn local features. Then, the extracted local features are input into bidirectional recurrent neural network (Bi-RNN) [36] modules to learn the relations between the different local features. Finally, following the Bi-RNN part is a dense layer used for regression. The details for every part of the model are given below.

The input for the model is a three-dimensional vector whose size is the batch size ×1000×2. The batch size is set to 64 in this study, which means that 64 cardiac cycles are input into the model to train at the same time. The other two dimensions are signal length and channel number (PCG and PPG). All the signals are zero-padded to 1 s before inputting to the model. 

The first residual convolutional block and the five subsequent blocks have almost the same structure, with differences in only some parameters’ values and with the beginning of the first block missing a rectified linear unit (ReLU) of the activation layer. As shown in Figure 3, the residual block is composed of two one-dimensional convolutional (Conv 1D) layers, followed by a batch normalization (BN) layer, a ReLU layer, and a dropout layer between two Conv 1D layers. There is also a max-pooling layer on another branch. As Table 2 shows, the pooling size and the stride of the second convolutional layer are 2 only in the 1st, 3rd, and 5th residual blocks, which reduces the length of the feature maps to half their original length in these blocks. In the other blocks, the pooling size is 1, which does not affect the length of the feature maps. Therefore, the output length of this part is 125 (1/8 of the input length).

As PCGs and PPGs (including learned feature maps) are strict time series data, recurrent neural networks (RNNs) with parameter-sharing structures are universally used to process these time sequences and memorize the context utilizing their internal state [28]. Here, a Gated Recurrent Unit (GRU) is selected as the implementation of an RNN, which offers the same performance as LSTM but with reduced training time [37]. The bidirectional layer is formed by two GRU layers in opposite directions: forward and backward. Then, the outputs of the two GRU layers are combined as the output features of the Bi-RNN layer. The Bi-RNN is followed by a BN, which is used to adjust and scale the inputs from former layers; then, a leaky version of the ReLU is utilized to avoid the vanishing gradient problem. In this study, the unit number is set to 32 for both the forward and backward GRU layers, producing an output feature vector length of 64. 

At the end of the model, a dense layer, which has 4 cells, is placed to output the estimated hemodynamic parameters and compare with the reference hemodynamic parameters using the loss function. As shown in Table 1, the min–max values are [50, 300], [−30, 20], [0, 10,000], and [−6000, 0] for SBP, DBP, MRR, and MRD, respectively, and SBP/50, DBP/10, MRR/2000, and MRD/1000 are considered as reference output values for the model to have the same contribution to the loss function. Then, the loss is used to update the parameters of the model via backpropagation. The learning rate for gradient descent is set to 0.001.

### 2.3. Hemodynamic Parameter Estimation Schemes

#### 2.3.1. Scheme I: Five-Fold Cross-validation of Recordings within Subjects

**Figure 4:**
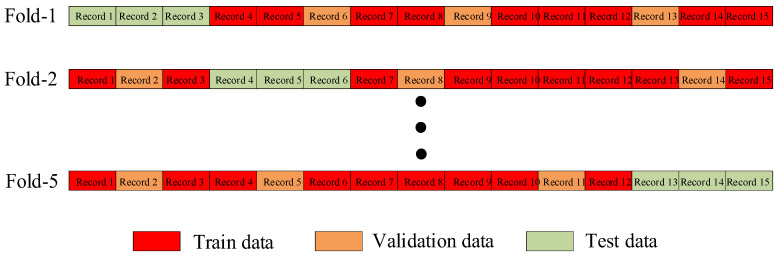
Data split diagram of 5-fold cross-validation for scheme I.

The records of each subject were randomly split into 5 parts to set up a 3-1-1 training, validation, and testing scheme of machine learning. For example, as Figure 4 shows, subject 1’s 15 records were divided into 5 equal subsets, each of which had 3 records. The first subset was used as test data, and the remaining 12 records were randomly split into training data and validation data in a 3:1 ratio. The model was evaluated on the validation set for 200 training epochs (an epoch refers to one cycle through the full training dataset), and the best validation model which had the smallest loss on the validation set was selected and evaluated on the test set to determine its performance. Then, this procedure was repeated on the other 4 subsets. In the end, all the records were tested once.

#### 2.3.2. Scheme II: Hemodynamic Parameter Estimation between Subjects

To assess the generalization of the model across subjects, one subject’s data were selected to evaluate the performance of the model trained on other subjects’ data. To see the performance on hidden test data, the target subject’s data were randomly divided into two equal subsets: one for validation and the other for testing. They were then swapped.

### 2.4. Performance Metrics

In this study, the Pearson correlation coefficient (CC), mean error (ME), mean absolute error (MAE), and standard deviation (SD) were used to evaluate performance. Their formulas are as follows:

(1) $$MAE=\frac{1}{N}\sum_{i=1}^{N}\left|{\hat{y}}_{i}-y_{i}\right|$$

(2) $$ME=\frac{1}{N}\sum_{i=1}^{N}\left({\hat{y}}_{i}-y_{i}\right)$$

(3) $$SD=\sqrt{\frac{1}{N-1}\sum_{i=1}^{N}{\left({\hat{y}}_{i}-y_{i}-ME\right)}^{2}}$$

(4) $$CC=\frac{\sum_{i=1}^{N}\left(y_{i}-\bar{y}\right)({\hat{y}}_{i}-\bar{\hat{y}})}{\sqrt{\sum_{i=1}^{N}{\left(y-\bar{y}\right)}^{2}}\sqrt{{\left({\hat{y}}_{i}-\bar{\hat{y}}\right)}^{2}}}$$

where $\hat{y}$ is the estimated value, *y* is the measured value, and *N* is the number of cardiac cycles.

## 3. Results

### 3.1. Results of Scheme I (within Subject)

**Figure 5:**
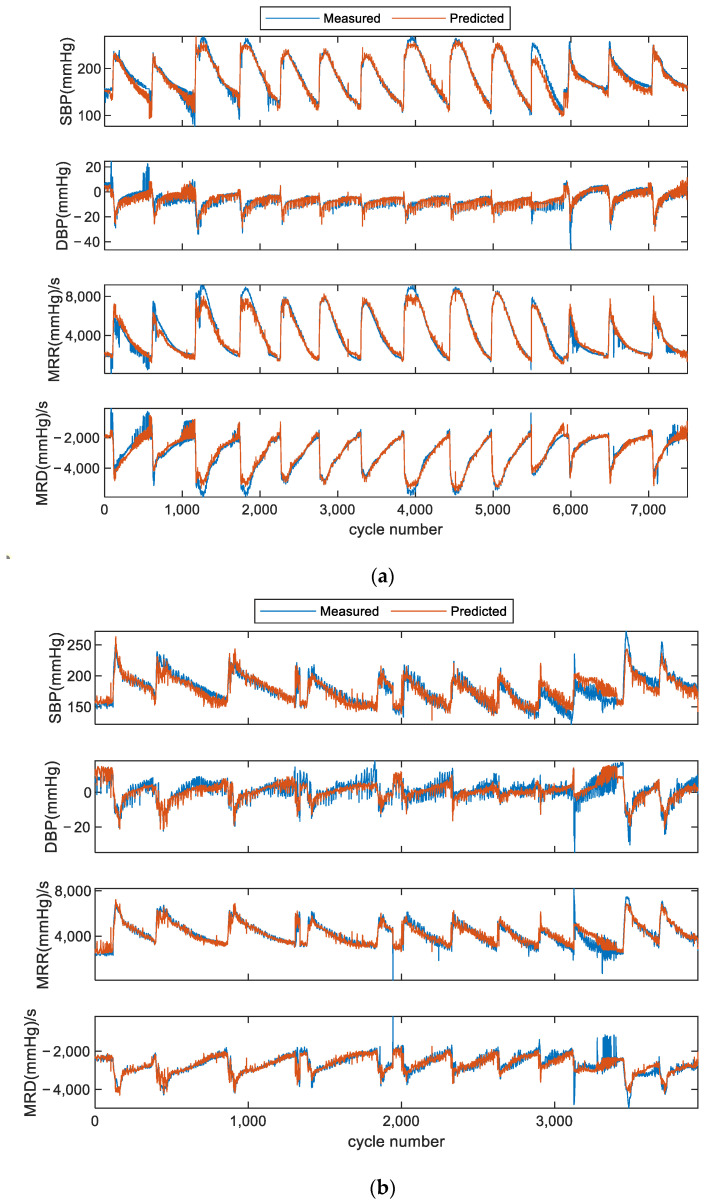
Comparison of estimated hemodynamic parameter values and measured values in scheme I. (**a**) Four examples of subject 1. (**b**) Four examples of subject 2.

**Figure 6:**
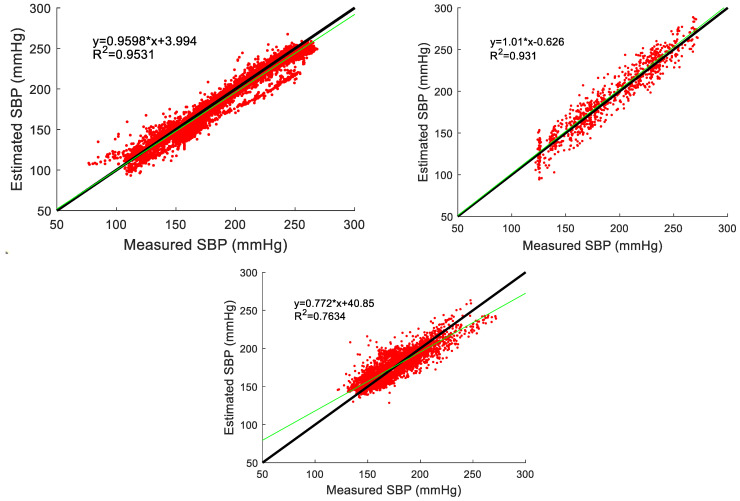
Correlation analysis between estimated and measured SBP in scheme I for all cycles of subject 1, subject 2, and subject 3.

As indicated in Figure 5, the estimated hemodynamic parameter values using the deep learning model were compared with the values extracted from blood pressure measurements taken with devices. Due to the limitation of the small number of cardiac cycles from subject 3, the performance was evaluated on subject 1 and subject 2. The changes in the parameters following the injection and metabolism of the drug are clearly shown in Figure 5. A strong correlation between the estimated values and measured values can be observed distinctly, and an example (subject 1’s SBP) of correlation analysis is shown in Figure 6. The results for SBP, DBP, MRR, and MRD are shown in Table 3. Six performance indicators (ME, MAE, SD, CC, P, and 95% CI for CC) for each of them were calculated. It was found that the proposed network predicted hemodynamics within subjects very accurately. The *p*-values in Table 3 show statistical significance, which were calculated using the estimated parameters and the corresponding measured values. Thus, the *p*-values prove whether the estimated and measured values were consistent or not.

### 3.2. Results of Scheme II (across Subjects)

**Figure 7:**
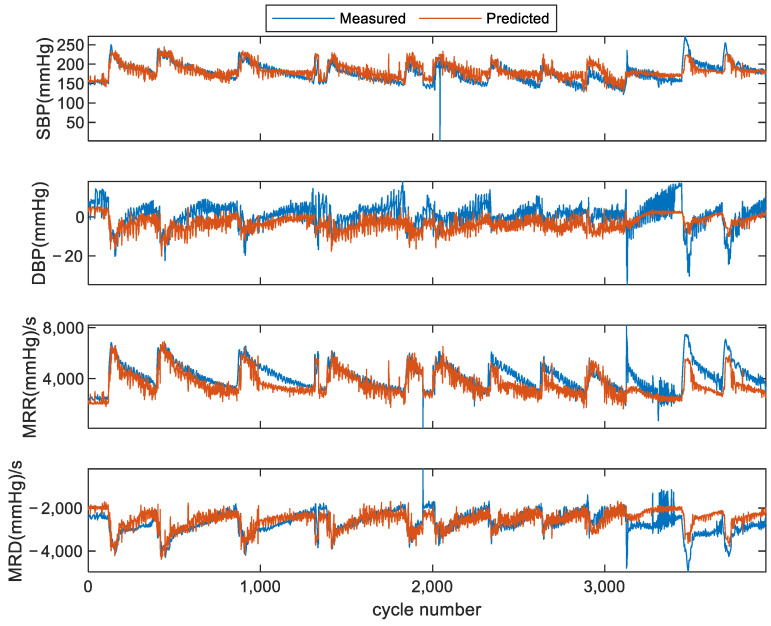
Comparison of estimated hemodynamic parameter values and measured values in scheme II for subject 2, where subject 1 and subject 3’s data were used as training data.

In this scheme, any two subjects’ data were used as training data, and the other subject’s data were selected as test data. The average results are shown in Table 4. An example (subject 2) of comparison between the measured values and estimated values in scheme II is shown in Figure 7. It can be seen that there were strong correlations between the estimated values and measured values, and there seemed to be a slight difference caused by individual differences. When using subject 1 and subject 3 to predict subject 2, the average MEs ± SDs were 7.9 ± 14.71 mmHg, −4.905 ± 4.605 mmHg, −419 ± 549 mmHg/s, and 73.5 ± 378 mmHg/s for SBP, DBP, MRR, and MRD, respectively, and the average CCs were 0.759, 0.556, 0.848, and 0.657. The average MEs ± SDs of subject 1 estimated using subject 2 and subject 3 were −0.675 ± 17.47 mmHg, 2.362 ± 5.595 mmHg, 147 ± 975.5 mmHg/s, and 226 ± 561 mmHg/s for SBP, DBP, MRR, and MRD, respectively, and the average CCs were 0.908, 0.715, 0.937, and 0.919.

### 3.3. Results with Calibration for Scheme II

**Figure 8:**
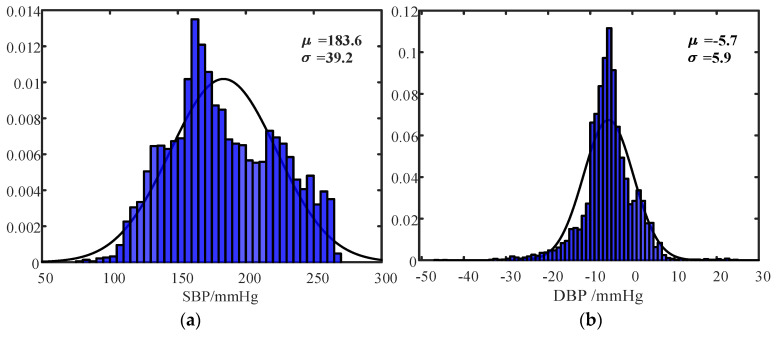

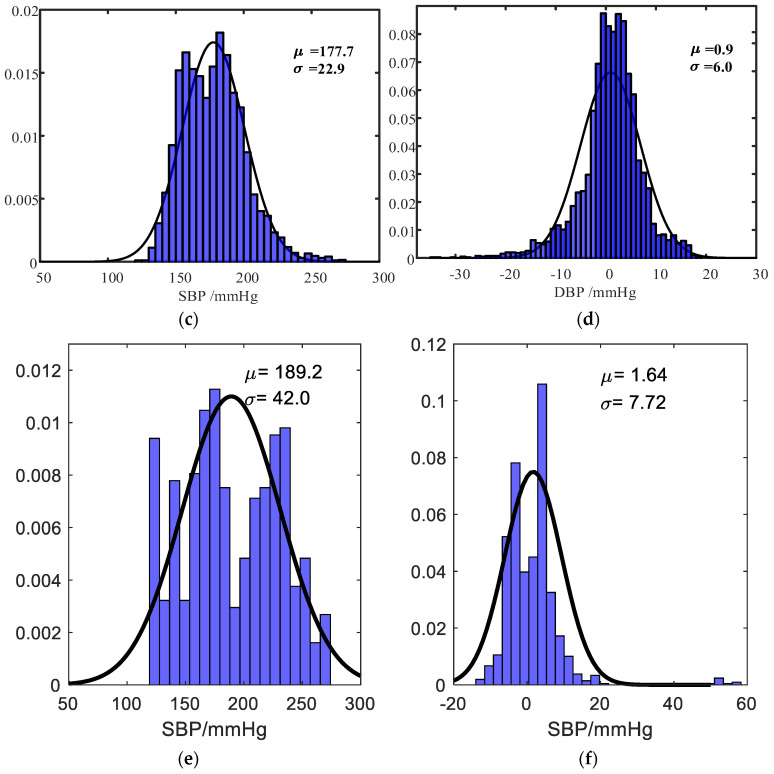
Probability density distribution. (**a**) SBP of subject 1; (**b**) DBP of subject 1; (**c**) SBP of subject 2; (**d**) DBP of subject 2; (**e**) SBP of subject 3; (**f**) DBP of subject 3.

The results across subjects also exhibited correlations, though the MAE and SD were much higher than those within a subject. The high errors were mostly caused by individual differences. Due to the limited number of subjects, individual differences cannot be eliminated. As the probability density shows in Figure 8, individual differences can be observed distinctly between subject 1 (Figure 8a,b) and subject 2 (Figure 8c,d).

**Figure 9:**
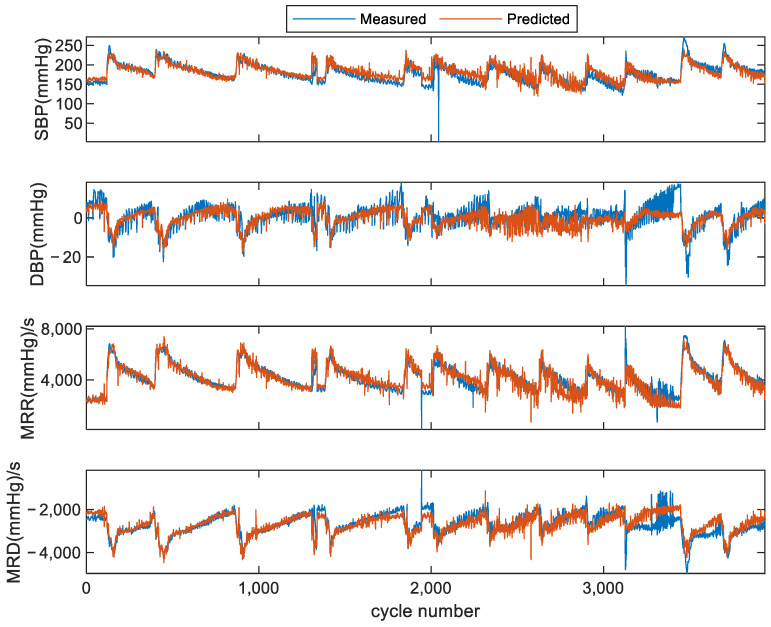
Comparison of estimated and measured hemodynamic values trained using one validation record for subject 2.

Next, to account for individual differences, we added a few records from the subject being evaluated to train the model. For example, when using subjects 1 and 3 to train the model and observing the performance on subject 2, the validation scheme was the same as scheme II, but one record from the validation data was also used to train the model. The model was evaluated on the remaining records, and test data were estimated to observe the performance. The comparison between the estimated and measured hemodynamic parameters of subject 2 after being calibrated with one record of subject 2 is shown in Figure 9. The average MEs ± SDs of subject 2 were 6.93 ± 12.595, −0.98 ± 3.995, 54 ± 446, and −37 ± 294 and the average CCs were 0.824, 0.674, 0.904, and 0.792 for SBP, DBP, MRR, and MRD, respectively. Similarly, Table 5 shows the results of subject 1 from the model trained with subjects 2 and 3. The results showed significant improvement and approached the results obtained within the same subject, which suggests that the model can be trained with existing data and adapted to monitor new patients using calibration methods to eliminate individual differences. Thus, further work should be conducted to study some calibration methods in the future.

## 4. Discussions

Previous research has studied the relationship between BP, PCGs, and PPGs, including cuff blood pressure estimation using PCGs [14], intra-arterial BP determination using PCGs [38], and BP prediction with PPGs [2]. Most previous works focused on feature analysis and BP estimation with the most relevant features that had high correlation coefficients with BP, and most previous models output one parameter (SBP or DBP) at a time, which is not enough for continuous hemodynamic monitoring. MRR and MRD, which are important indicators for cardiac systolic and diastolic functions, have seldom been estimated in such studies. In this study, a model based on deep learning was built to learn deep nonlinear features from PCG and PPG signals, and it is the first to simultaneously estimate four hemodynamic parameters on a beat-by-beat basis: SBP, DBP, MRR, and MRD. The data used in this study were from anesthetized beagle dog experiments, with a larger range of BP than in previous studies. The ranges of SBP, DBP, MRR, and MRD were 77–272 mmHg, −47–20 mmHg, 106–9181 mmHg/s, and −5896–70 mmHg/s, respectively.

The within-subject results showed that there are strong correlations between the measured and estimated hemodynamic parameters. The CCs of all parameters validated by subject 2 were lower than those for subject 1 due to low signal quality. For subject 1, the percentages of MAEs and SDs accounting for the range of parameters were 3.3%, 2.7%, 3.8%, and 3.2% for SBP, DBP, MRR, and MRD, respectively. Meanwhile, for subject 2, the percentages of MAEs and SDs accounting for the range of parameters were 5.4%, 5.2%, 3.3%, and 3.5% for SBP, DBP, MRR, and MRD. As we performed testing with records not used for training the model, it shows good potential application for monitoring intraventricular hemodynamic parameters even in home settings using non-invasive methods after collecting data for one specific patient, whose data range will be much smaller than what was used in this study.

As few studies have estimated LVDBP, LV ± dP/dtmax, only SBP results were compared with previous studies, as shown in Table 6. The values in this table are reported directly from the original articles. Peng et al. [14] predicted cuff BP with a regression model using the Fourier spectrum of the second heart sound. Tenfold cross-validation was performed for each subject. The average MAE and SD of SBP were 4.339 and 6.121 mmHg, which are higher than those in our study. Unlike the scheme of splitting data used in this study, the sample data of one subject were randomly split into training data and test data; therefore, nearby samples in the test data may have been involved in training data. In this way, the high performance may be less convincing for practical application. Tang et al. [20] extracted multi-domain features from PCGs to estimate intraventricular SBP and analyzed the relationships between SBP and different features. The average MAE and SD were 6.86 and 8.96 mmHg, which are similar to the SBP estimation results in this work, even though 10-fold cross-validation was performed on each record, whose test samples and training samples were very similar in their work. Kapur et al. [38] estimated inter-arterial (lower limbs) SBP and DBP using features of PCGs and artificial neural networks with cuff BP-based regularization, and data were obtained from 25 patients with artificial heart valves. The samples were randomly split without considering subject information, and the SBP results showed an RMSE of 7.305 mmHg and SD 7 of mmHg, a performance close to that in this work. Esmaelpoor et al. [39] proposed an architecture with two neural networks to predict SBP and DBP using PPG signals. For SBP, an MAE of 3.97 mmHg and SD of 5.55 mmHg were reported with the SBP range of 80–160 mmHg. Yan et al. [21] developed a deep convolutional neural network to estimate SBP and DBP simultaneously. The MAE and SD for SBP were 3.09 and 2.76 mmHg when randomly splitting all subjects’ samples, which are better results than those we achieved. However, we took different time period information into account by avoiding the use of nearby samples of test data to train the model.

The current study has some limitations: (1) the dataset is limited, as both the number of subjects and the number of records are very limited. As a consequence, the network is not sufficiently trained. (2) The network architecture is somewhat complicated. Much time is needed to train the network. A lightweight network is expected in the future to complete this task. (3) The trained network has not been tested with human data. The generalization needs further validation across different datasets. 

## 5. Conclusions

In this study, we built a deep learning model to predict four intraventricular hemodynamic parameters using non-invasive apparatus (PCGs and PPGs). The model combines a residual convolutional module and a Bi-GRU module which learn the local features and context relations, respectively. The fivefold validation over different records within subjects showed that there were high correlations between PCGs, PPGs, and hemodynamic parameters at different times for a given subject. Given the large range of BP induced by epinephrine investigated in this study, prediction errors in a normal range of data are expected to be much smaller than those observed here. It was also shown that the high error between subjects could be calibrated by adding some individual information. Therefore, more studies about calibration methods should be conducted. The practical application of the algorithm for home healthcare and real-time BP monitoring could be realized with the expeditious development of wearable devices.

## Figures and Tables

**Table 1:** Details of the hemodynamic parameters extracted from records.

	Record Index	No. of Cardiac Cycles	SBP (mmHg) (Min–Max)	DBP (mmHg) (Min–Max)	MRR (mmHg/s) (Min–Max)	MRD (mmHg/s) (Min–Max)
Subject 1	1	72	[128, 158]	[2, 8]	[1812, 1981]	[−1998, −1900]
	2	567	[115, 231]	[−22, −4]	[1452, 7595]	[−4795, −1450]
	3	587	[106, 268]	[−25, −3]	[1467, 9080]	[−5781, −1546]
	4	563	[112, 264]	[−22, −3]	[1430, 8946]	[−5756, −1437]
	5	523	[101, 252]	[−22, −3]	[481, 8564]	[−5431, −365]
	6	419	[102, 254]	[−26, −6]	[1329, 7910]	[−4501, −1524]
	7	512	[138, 258]	[−47, 9]	[1383, 6522]	[−4633, −1722]
	8	574	[152, 258]	[−30, 6]	[1749, 6496]	[−4715, −1699]
	9	533	[157, 250]	[−28, 5]	[1841, 6312]	[−5031, −1116]
	10	502	[153, 238]	[−29, 2]	[107, 5778]	[−4321, −70]
	11	558	[97, 234]	[−18, 7]	[1732, 7502]	[−5140, −855]
	12	578	[77, 268]	[−34, 10]	[1810, 9181]	[−5896, −859]
	13	510	[92, 264]	[−33, 2]	[1711, 8946]	[−5845, −1010]
	14	503	[122, 239]	[−26, 0]	[1535, 7942]	[−5041, −1539]
	15	528	[117, 241]	[−25, −3]	[1465, 7981]	[−5070, −1503]
Subject 2	16	118	[147, 160]	[−1, 14]	[2258, 273]	[−2552, −2166]
	17	142	[144, 212]	[−14, 18]	[106, 6000]	[−3820, −175]
	18	341	[133, 218]	[−10, 12]	[1849, 6137]	[−3746, −1680]
	19	315	[137, 224]	[−8, 14]	[2664, 6097]	[−3654, −1676]
	20	272	[131, 213]	[−7, 11]	[2230, 5743]	[−3451, −1782]
	21	247	[122, 194]	[−7, 10]	[2594, 5624]	[−3390, −1384]
	22	287	[141, 235]	[−35, 17]	[699, 8198]	[−4793, −1143]
	23	275	[155, 272]	[−30, 17]	[2371, 7470]	[−4973, −2336]
	24	254	[164, 255]	[−24, 10]	[2753, 7091]	[−4257, −2287]
	25	270	[166, 250]	[−20, 9]	[3362, 6833]	[−4128, −2158]
	26	439	[162, 239]	[−22, 9]	[3293, 6731]	[−4290, −1974]
	27	472	[158, 231]	[−20, 9]	[3221, 6567]	[−4190, −1806]
	28	38	[149, 221]	[−17, 15]	[3094, 6137]	[−3847, −1799]
	29	465	[148, 218]	[−15, 14]	[3021, 6183]	[−3904, −1776]
Subject 3	30	50	[122, 138]	[4, 8]	[849, 1205]	[−1242, −1051]
	31	164	[137, 255]	[−12, 11]	[6, 7896]	[−3838, −189]
	32	107	[140, 248]	[−12, 12]	[−53, 7258]	[−3693, −97]
	33	85	[137, 236]	[−11, 20]	[−48, 6828]	[−3496, −238]
	34	42	[142, 231]	[−11, 17]	[2, 6705]	[−3159, 4]
	35	85	[124, 200]	[−5, 7]	[−35, 5717]	[−3330, 0]
	36	71	[134, 217]	[−3, 15]	[100, 5035]	[−2784, −20]
	37	49	[137, 202]	[−1, 8]	[160, 4247]	[−1950, −32]
	38	90	[164, 272]	[−11, 12]	[136, 8019]	[−4104, −212]
	39	118	[159, 266]	[−11, 12]	[−442, 8319]	[−3674, −246]
	40	3	[235, 263]	[−12, −8]	[7237, 8089]	[−3673, −343]
Total		12,328	[77, 272]	[−47, 20]	[106, 9181]	[−5896, −70]

**Table 2:** The details of the parameters in residual blocks.

Residual Blocks	No. of Conv	Kernel Length	Kernel Number	Stride of Conv	Pooling Size
1st	#1	16	32	1	2
	#2	16	32	2	
2nd	#1	16	32	1	1
	#2	16	32	1	
3rd	#1	16	32	1	2
	#2	16	32	2	
4th	#1	16	32	1	1
	#2	16	32	1	
5th	#1	16	64	1	2
	#2	16	64	2	
6th	#1	16	64	1	1
	#2	16	64	1	

**Table 3:** Performance metrics of scheme I (within subject).

Performance Metrics	Subject 1			
	SBP (mmHg)	DBP (mmHg)	MRR (mmHg/s)	MRD (mmHg/s)
ME	−3.41	−0.1	−39	59
MAE	6.22	1.54	329	175
SD	6.69	2.42	428	226
CC	0.984	0.916	0.979	0.972
*p*-value	<<0.001	<<0.001	<<0.001	<<0.001
95% CI for CC	0.982–0.985	0.908–0.924	0.977–0.981	0.969–0.975
Performance metrics	Subject 2			
	SBP (mmHg)	DBP (mmHg)	MRR (mmHg/s)	MRD (mmHg/s)
ME	−0.12	−0.4	37	−0.9
MAE	8.23	2.77	267	169
SD	9.97	3.59	383	546
CC	0.897	0.812	0.922	0.873
*p*-value	<<0.001	<<0.001	<<0.001	<<0.001
95% CI for CC	0.883–0.910	0.787–0.834	0.911–0.932	0.855–0.888
Performance metrics	Subject 3			
	SBP (mmHg)	DBP (mmHg)	MRR (mmHg/s)	MRD (mmHg/s)
ME	3.81	0.20	48.8	−49.2
MAE	6.82	1.94	389	215
SD	6.99	2.92	468	446
CC	0.923	0.856	0.939	0.886
*p*-value	<<0.001	<<0.001	<<0.001	<<0.001
95% CI for CC	0.921–0.942	0.848–0.864	0.927–0.941	0.869–0.895

**Table 4:** Performance metrics of scheme II (between subjects).

	Performance Metrics	SBP (mmHg)	DBP (mmHg)	MRR (mmHg/s)	MRD (mmHg/s)
Subject 1	ME	−0.675	2.365	147	226
	MAE	13.14	5.04	776.5	414
	SD	17.47	5.595	975.5	561
	CC	0.908	0.715	0.937	0.919
	*p*-value	<<0.001	<<0.001	<<0.001	<<0.001
	95% CI for CC	0.903–0.914	0.699–0.731	0.933–0.941	0.914–0.924
Subject 2	ME	7.895	−4.905	−419	73.5
	MAE	12.52	5.74	541	314
	SD	14.71	4.605	549	378
	CC	0.759	0.556	0.848	0.657
	*p*-value	<<0.001	<<0.001	<<0.001	<<0.001
	95% CI for CC	0.740–0.776	0.526–0.586	0.836–0.860	0.632–0.681
Subject 3	ME	−0.386	−2.895	−234	−236
	MAE	13.90	5.09	792	412
	SD	18.41	5.260	950	526
	CC	0.885	0.693	0.903	0.862
	*p*-value	<<0.001	<<0.001	<<0.001	<<0.001
	95% CI for CC	0.870–0.896	0.686–0.706	0.896–0.910	0.852–0.871

**Table 5:** The calibration performance using one record of the target subject.

	Indicators	SBP (mmHg)	DBP (mmHg)	MRR (mmHg/s)	MRD (mmHg/s)
Subject 1	ME	9.06	0.32	153	150
	MAE	14.180	3.970	773	379
	SD	14.34	5.00	904.00	541.50
	CC	0.94	0.72	0.95	0.94
	*p*-value	<<0.001	<<0.001	<<0.001	<<0.001
	95% CI for CC	0.934–0.942	0.705–0.736	0.949–0.955	0.931–0.939
Subject 2	ME	6.93	−0.98	54	−37
	MAE	10.67	3.06	335	222
	SD	12.595	3.995	446	294
	CC	0.824	0.674	0.904	0.792
	*p*-value	<<0.001	<<0.001	<<0.001	<<0.001
	95% CI for CC	0.809–0.837	0.650–0.696	0.896–0.912	0.776–0.807
Subject 3	ME	8.43	0.68	83	67
	MAE	12.87	4.46	547	306
	SD	13.45	4.85	679	436
	CC	0.854	0.71	0.913	0.832
	*p*-value	<<0.001	<<0.001	<<0.001	<<0.001
	95% CI for CC	0.849–0.867	0.696–0.721	0.906–0.922	0.826–0.847

**Table 6:** Comparison with published studies.

References	Signal Sources	BP Type	Method	SBP Range (mmHg)	Performance	Performance Account for SBP Range
Tang et al. [18], 2017	PCG	Left ventricular BP	Multi domain feature +SVM	same in this study	CC: 0.92 MAE: 6.86 mmHg SD: 8.96 mmHg	MAE: 3.5% SD: 4.6%
Peng et al. [14], 2015	PCG	Finger cuff BP	Fourier spectrum of second heart sound +SVM	about 90–140	CC: 0.707 MAE: 4.339 mmHg SD:6.121 mmHg	MAE: 8.6% SD: 12.2%
Kapur et al. [38], 2019	PCG	Intra-arterial BP	Characteristics of S1 and S2 +ANN	58–173	1. Without regularization: CC: 0.679 RMSE: 20.408 mmHg SD: 20 mmHg 2. Cuff BP regularization: CC: 0.964 RMSE: 7.305 mmHg SD: 7 mmHg	1. RMSE: 17.7% SD: 17.4% 2. RMSE: 6.3% SD: 6.1%
Esmaelpoor et al. [39], 2020	PPG	Invasive BP	Deep neural network	80–180	MAE: 3.97 mmHg SD: 5.55 mmHg	MAE: 4.0% SD: 5.6%
Yan et al. [21], 2019	PPG + ECG	Arterial BP	Deep CNN	80–180	1. Random split all subjects’ samples: MAE: 3.09 mmHg SD: 2.76 mmHg 2. Between subjects: MAE: 12.49 mmHg SD: 9.43 mmHg	1. MAE: 3.1% SD: 2.8% 2. MAE: 12.5% SD: 9.4%
Current study	PCG + PPG	Left ventricular BP	Deep learning model	77–272	1. Within subject: CC: 0.94 MAE: 7.23 mmHg SD: 8.33 mmHg 2. Between subjects: MAE: 12.8 mmHg SD: 16.1 mmHg	1. MAE: 3.7% SD: 4.3% 2. MAE: 6.6% SD: 8.3%

Note: SVM, support vector machine; ANN, artificial neural network; RMSE, root mean square error; CNN, convolutional neural network.

## Data Availability

The data are available on request.
